# The transcriptome of dendritic cells redraws the boundaries between pathogenicity and commensalism in yeast

**DOI:** 10.15698/mic2026.07.882

**Published:** 2026-07-07

**Authors:** Lisa Rizzetto, Irene Stefanini, Stefano Nenciarini, Monica Di Paola, Samantha Riccadonna, Ivo G. Gut, Marta Gut, Simon Heath, Duccio Cavalieri

**Affiliations:** 1 Fondazione Edmund Mach, San Michele all’Adige, Trento, 380980, Italy; 2 Department of Life Sciences and Systems Biology, University of Turin, Turin, 10123, Italy; 3 Department of Biology, University of Florence, Florence, 50100, Italy; 4 CNAG-CRG, Centre for Genomic Regulation (CRG), Barcelona Institute of Science and Technology (BIST), Barcelona, 08003, Spain; 5 Universidad Pompeu Fabra (UPF), Barcelona, 08002, Spain

**Keywords:** fungi, yeast, dendritic cells, non-pathogenic, pathogenic, phagolysosome, transcriptomics

## Abstract

Our immune system is constantly exposed to fungi, but it mounts a consistent, disease-related, and species-specific inflammatory response only against a few fungal species. Most of the current understanding of fungus-host interactions is based on a limited number of strains, hence neglecting the fungal intra-specific genetic and phenotypic diversity. To expand our knowledge of the spectrum of immune responses to pathogenic and non-pathogenic fungi, we compared the cytokine and transcriptional profiles of human monocyte-derived dendritic cells exposed to *Aspergillus fumigatus*, *Candida albicans*, *Candida parapsilosis*, and *Saccharomyces cerevisiae* strains. The tested species triggered common and species-specific responses, mostly resulting from the different timing of signaling pathways. Faster phagolysosome acidification was observed for pathogenic species. These results highlight the urgency to redraw the boundaries between pathogenicity and commensalism in fungi, shining a spotlight on the timing of the response, rather than solely on the genes triggered by the stimuli.

## INTRODUCTION

Our constant exposure to commensal and environmental fungi requires a competent immune system prone to activate tolerance, protective, and/or clearance mechanisms while avoiding collateral damage caused by excessive inflammation. Innate responses to fungal pathogens are initiated by antigen-presenting cells, like dendritic cells (DCs), upon recognition of fungal components via an array of microbial-associated receptors 1–5. The innate recognition of fungi is based on the ability of immune receptors to bind specific fungal wall components (e.g. mannans, β -glucans, β -mannosides) or nucleic acids and start a cascade of events that will result in the expression of inflammatory cytokines and chemokines, driving recruitment and activation of innate phagocytic cells to the site of infection and priming the appropriate T cell responses 6–8. Different signaling pathways can lead to the same outcome, which is in turn decided by a finely tuned balance between different signals 9, 10. This complex of signals is highly susceptible to small variations, resulting in the immune system responding differently to different forms of the same or different strains of the same species 11–16. Yeast-mediated DC maturation was demonstrated for different yeasts, including *Saccharomyces cerevisiae*, *Schizosaccharomyces pombe*, and *Candida albicans* 17–19, and cytokine secretion by DC in response to fungi was shown to be a tangled task, varying from one fungal stimulus to another 20–24, also depending on pathogen size 25. Considering all these observed differences in the response to co-specific fungal strains, categorizing the immune responses according to the fungal species seems to be an excessive simplification of the real situation, as it would imply generalizing to the entire species what is observed for a single strain. Yet, our current knowledge is mainly based on results obtained on a small number of strains representative of the fungal species 12–16. In addition to limiting our ability to assess the intra-species biodiversity, the use of a few strains, often exacerbating the species characteristics, has also limited our ability to estimate the overlap of immune responses to different (pathogenic and non-pathogenic) fungi. To properly combat fungal infection, a sophisticated understanding of the molecular mechanisms involved in pathogen recognition and host immune response is required. Transcriptomics can help in this intent, as this approach has become a key tool in deciphering the molecular changes of immune cells accompanying fungal infections 9, 26–28. We used RNA-sequencing to investigate both common and species-specific immune responses to fungi by challenging DCs with different *Candida albicans* (*Cal*), *Candida parapsilosis* (*Cpa*), *Aspergillus fumigatus* (*Afu*), and *Saccharomyces cerevisiae* (*Sce*) strains, revealing how different fungi demand specific DC transcriptional responses for their elimination or tolerance.

## RESULTS

### Dendritic cells show common and species-specific responses to pathogenic and non-pathogenic fungi

To fully explore the diversity of immune responses triggered by pathogenic and non-pathogenic fungi, we exposed DCs obtained from multiple donors to a selected set of fungal strains. Strain selection included a range of pathogenic fungal species causing life-threatening infections, including *Candida albicans* (*Cal*), *Candida parapsilosis* (*Cpa*), and *Aspergillus fumigatus* (*Afu*), and the GRAS (Generally Recognized As Safe) species *Saccharomyces cerevisiae* (*Sce*). For each species, multiple strains were selected to cover a broad range of intra-specific variability in terms of inflammatory potential (Table S1). Three strains were selected for the *Afu* species: AF293 (wild type), AF293 VKBR1, a color mutant (brown) causing a limited induction of IL-10 production and hence a moderate inflammation, and AF293 VKWH2, a color mutant (white) inducing a high level of IL-10, resulting in TH2 response and hence in the worst infection outcome in mice 14. Three strains were also selected for the *Cal* species, including the SC5314 reference strain, which produced low inflammation rates when administered to mice, the t1.1 mutant, including a copy of Leu tDNACAGLeu gene into the RPS10 genome locus resulting in Leu misincorporation at CUGs and hence in a severe immune response (higher production of TNF-alpha and IL-17A than the reference strain), and the mutant t2.1, including two copies of the same gene and associated with a more severe immune response compared to the one triggered by the t1.1 strain 29. Two clinical strains were included for the *Cpa* species: YB1, characterized by the induction of a moderate TH17 response, and YA4, characterized by an inflammation-priming of Treg response by induction of IL-10 30. Concerning the GRAS species, *Sce*, a broader range of strains were included with variable capabilities to promote diverse immune responses: the laboratory strain SK1 and the clinical YB7 strain, inducing IL-10-derived Th responses, the clinical strain Y13EU promoting high TNF-alpha and IL-6, the YA5 strain, inducing a moderate inflammation with fungal cells clearance, and the wine fermentation BB1533 strain, inducing a moderate inflammatory response characterized by a moderate induction of IFN-gamma and IL-17 30.

**Figure 1:**
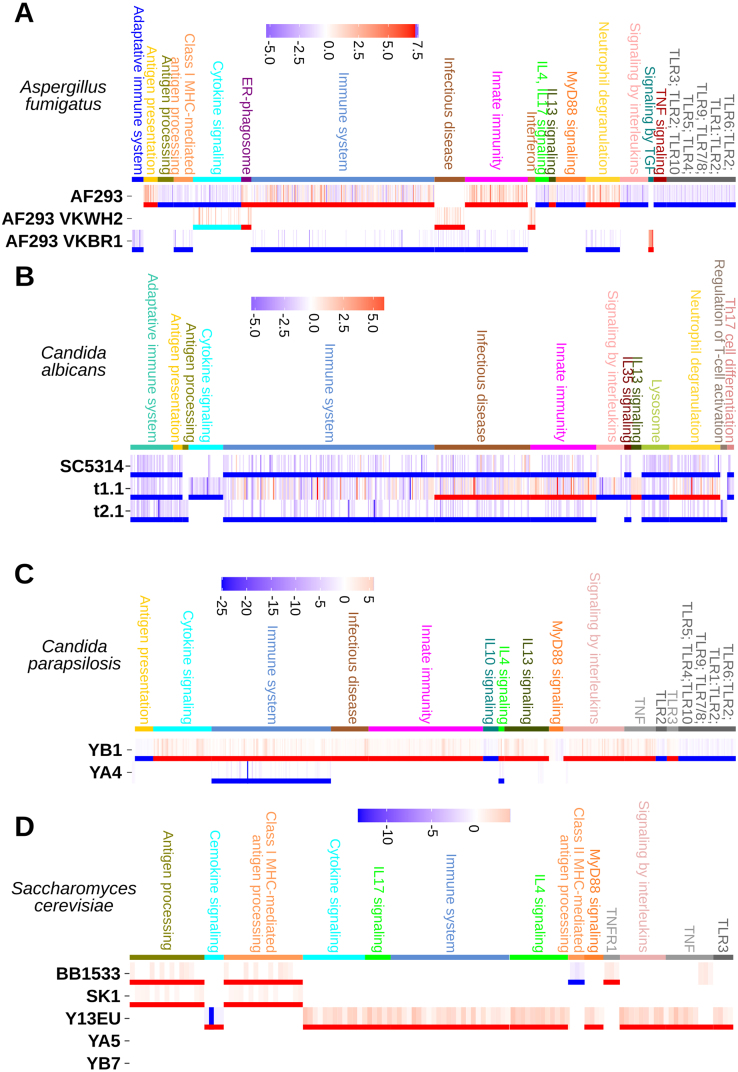
Intra-specific comparison of enriched pathways triggered by the exposure of Dendritic Cells with different strains. Comparison of pathways differentially represented in different *Aspergillus fumigatus***(A)**, *Candida albicans***(B)**, *Candida parapsilosis***(C)**, and *Saccharomyces cerevisiae***(D)** strains. Data show the Log2(Fold Change) values for genes differentially expressed (DEGs), and colored according to the color legend, in the strain indicated in the left part of the plot and the pathways (KEGG or Reactome) differentially represented specifically for the listed strains. Red and blue horizontal bars at the bottom of each line showing the DEGs indicate if the pathway is over- or underrepresented, respectively.

The comparison of the transcriptional profiles of DCs exposed to different co-specific strains highlighted relevant information on the nuanced molecular mechanisms dictating the outcome of host-fungal interactions. To address this, we focused on genes significantly differentially expressed (DEGs) in a single strain of each species (Table S2). Within *Aspergillus fumigatus*, the wild-type strain (AF293) specifically triggered pathways for antigen processing and cross-presentation (Figure 1**A**), supported by a robust upregulation of the genes VAMP8 (Log2FC = 3.31; p-adj < 0.001) and SEC22B (Log2FC = 2.18; p-adj < 0.001)(Table S2). Conversely, the VKWH2 mutant failed to activate these hubs and instead exhibited a unique under-representation of Toll-Like Receptor (TLR5 and TLR7/8) cascades (Figure 1**A**) through the downregulation of mediators such as TRAF6 (Log2FC = −1.62; p-adj < 0.01) and MAPK14 (Log2FC = −1.58; p-adj < 0.01)(Table S2). This divergence aligns with the known role of SEC22B and VAMP8 in orchestrating effective fungal phagosome maturation and antigen presentation in DCs 31, 32, suggesting that VKWH2 actively ‘silences’ the machinery required for a protective Th1 response. A similar contrast emerges in *Candida albicans*, where the transcriptomic fingerprint of the reference strain SC5314 reflects its high pathogenic potential through the exclusive induction of genes related to lysosomal biogenesis and N-glycan trimming (Figure 1**B**), such as LAMP1 (Log2FC = 2.84; p-adj < 0.001), GLB1 (Log2FC = 3.12; p-adj < 0.001), and CANX (Log2FC = 2.35; p-adj < 0.001) (Table S2), indicating superior metabolic readiness and phagocytic activity. In contrast, the clinical isolate T2.1 shifts the profile toward an induction of pathways to reduce DC fitness and evade immune surveillance (Figure 1**B**). These findings support the hypothesis that highly virulent strains like SC5314 prioritize rapid lysosomal remodeling to survive the intracellular environment 33, while other isolates may favor DC function impairment as an immune evasion strategy. The response to *C. parapsilosis* reveals a unique lipid metabolic checkpoint (Figure 1**C**). Transcriptomic data for the YB1 isolate show a significant under-representation of fatty acid biosynthesis and metabolism. This is driven by the robust downregulation of key enzymatic hubs, including SCD (Log2FC = −1.78; p-adj < 0.01), FADS1 (Log2FC = −1.65; p-adj < 0.01), and ELOVL5 (Log2FC = −1.52; p-adj < 0.05)(Table S2). This metabolic rewiring suggests that *C. parapsilosis* modulates DC membrane lipid composition to “tune” the efficiency of TLR-mediated recognition 34, explaining the balanced, intermediate cytokine profile observed in our functional assays. Finally, the response to *S. cerevisiae* strains showed a precise gradient where opportunistic strains (e.g., Y13EU) specifically target the Interleukin-6 family signaling and MyD88-independent TLR4 cascades (Figure 1**D**) marked by the significant upregulation of SOCS3 (Log2FC = 2.45; p-adj < 0.01) and OSM (Log2FC = 2.31; p-adj < 0.01)(Table S2). This distinguishes them from industrial strains, which remain focused on basal MHC-II presentation (Figure 1**D**). This gradient highlights how opportunistic evolution fine-tunes the fungal-DC interface, evolving from simple commensal recognition to a more complex immune-modulatory profile. The specific recruitment of the SOCS3-OSM axis 35 by opportunistic *S. cerevisiae* highlights a transition toward a more complex immune-modulatory profile, prone to persistence within the gut environment, typical of yeasts capable of colonizing the host.

**Figure 2:**
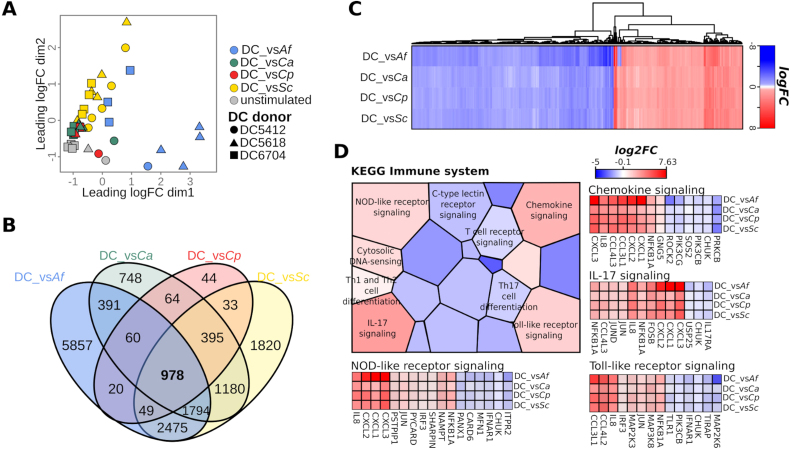
Transcriptional analysis of Dendritic Cells challenged with strains of various fungal species. **(A)** First two components resulting from the MDS (MultiDimensional Scaling) carried out on the lists of DEGs (Differentially Expressed Genes, fdr < 0.05) of DCs challenged with *A. fumigatus*, *C. albicans*, *C. parapsilosis*, and *S. cerevisiae* compared to unchallenged DCs. **(B)** Venn diagram comparing the lists of DEGs resulting from the DC challenge with fungal species. **(C)** Heatmap showing, for each fungal species, the log2FC values of genes differentially expressed in every DC challenge with fungal species. **(D)** Voronoi tessellation representation of the KEGG Immune system pathways differentially represented in DC exposed to the tested fungi. Colors report the average expression of every annotated gene for the corresponding term as indicated in the embedded legend. Heatmaps report the expression level of the genes involved in the indicated pathways. Voronoi tessellation and drawing were carried out with Voronto. DC_vsAfu = Dendritic Cells challenged with *A. fumigatus* strains, DC_vsCal = Dendritic Cells challenged with *C. albicans* strains. DC_vsCpa = Dendritic Cells challenged with *C. parapsilosis* strains, DC_vsSce = Dendritic Cells challenged with *S. cerevisiae* strains.

MultiDimensional Scaling (MDS) on the number of reads identified for each gene in all the treatments (Figure 2**A**) revealed that DCs transcriptional profiles differentiated according to the challenged species but not according to the DC donor (permutational multivariate analysis of variance p = 0.003 and 0.967, respectively). A separation of DCs treated with *Afu* strains (DCs_vs*Af*) over the first dimension and of DCs challenged with *Sce* strains (DCs_vs*Sc*) over the second dimension was clear (Figure 2**A**). Prompted by the lack of significant differences among the responses induced in DCs from different donors and by the significant discrimination of the profiles induced by different fungal species, we proceeded with the analysis of the immune response induced by the different species, including all the differential immunogenic impact of the co-specific strains. With this intent, we identified the DEGs as a consequence of the DC exposure to co-specific strains (Table S3). By comparing the list of DEGs, we found 978 genes differentially expressed in all the challenges with fungal species (“shared DEGs”, Figure 2**B**). Shared DEGs had similar expression profiles across DCs, apart from 12 genes (*BATF3*, *ESPNL*, *KAT5*, *MFSD10*, *MFSD12*, *NABP1*, *SHB*, *SLC15A3*, *TRABD*, and 3 uncharacterized genes), which were under-expressed in DCs_vs*Af* and over-expressed in response to the other fungal species (Figure 2**C** and Table S4). Voronoi tessellation on shared DEGs highlighted changes in many pathways related to immunity, with genes involved in chemokine, IL-17, Toll-like receptor, and NOD-like receptor signaling, as well as cytosolic DNA-sensing being on average over-expressed in the challenges (Figure 2**D**). Pathways and GOs enrichment analysis further highlighted similarities among transcriptomic profiles (Figure S1 and Table S4). Every fungal species induced in DCs an over-representation of chemokine receptors binding chemokines, cytokine-cytokine receptor interaction (Figure S2), TNF (Figure S3), and MAPK signaling (Figure S4) pathways (Table S3). The Toll-like receptor pathway was differentially represented in DC_vs*Af* and DC_vs*Cp* (DCs challenged with *Afu* or *Cpa* strains) only, with an over-expression of genes inducing pro-inflammatory (DC_vs*Af*) and chemotactic effects (DC_vs*Af* and DC_vs*Cp*) (Figure S5). DCs exposed to *Afu* and *Cal* strains showed an over-representation of the cell adhesion molecules pathway, with an over-expression of *MHC-I*, *MHC-II* (DC_vs*Af* and DC_vs*Ca*), and CD80 (DC_vs*Ca*), and an under-expression of *PD-L2* (DC_vs*Af* and DC_vs*Ca*) (Figure S6). The inflammatory bowel disease (IBD) pathway was found to be over-represented in DC_vs*Af* and DC_vs*Sc* DEGs (Table S4 and Figure S7), in line with the enrichment of *Sce* strains reported in fecal samples from IBD children 16, 36.

### Different timing of signaling pathways characterizes DC species-specific response to fungi

**Figure 3:**
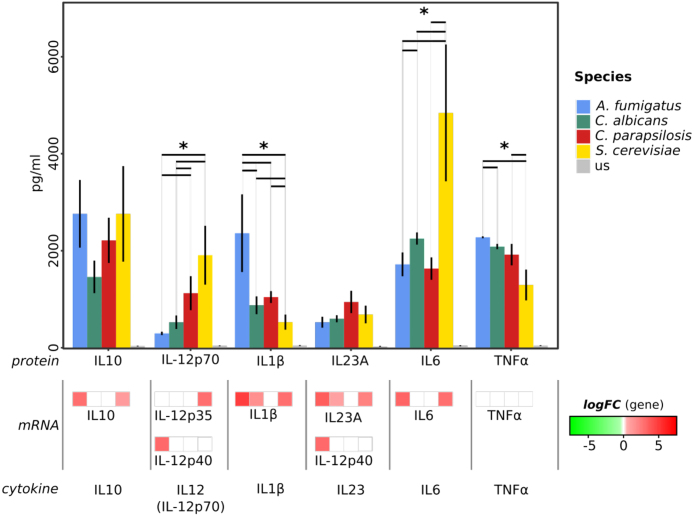
Quantification of cytokines produced by DCs challenged with strains of four fungal species. Histograms show the average value of cytokine levels measured in three independent (different DC donors) replicates. Error bars indicate standard errors. The Wilcoxon signed-rank test was performed to assess significant differences among cytokine levels produced by DCs challenged with the tested fungal species. * = p < 0.05.

As expected, according to the samples’ distribution in the MDS based on the lists of DEGs (Figure 2**A**), *Afu* strains induced the most different response in DCs, with 5857 DEGs specific for this challenge (Figure 2**B**). Several pathways relevant to the immune response to microbes were differentially represented specifically in the challenge with *Afu* strains: among these, chemokine signaling (Figure S8) and IL-6 signaling pathways (Figure S9 and Table S5). The IL-6 signaling pathway was found to be an early but transient response, as after 4 hours of DC challenge with the fungus, the IL-6 gene was overexpressed in DC_vs*Afu* but genes involved in IL-6 signaling were mainly under-expressed (Figure S9), and the amount of IL-6 produced by DCs after 24 hours was lower than in response to *Sce* and *Cal* (Figure 3). Previous studies have shown that the activation of the IL-6 signaling pathway, as a proxy of phagolysosome activation 37, could be due to different timing in acidification of the phagolysosome 12, and this trend was also confirmed at the transcriptional level (Figure S10). By annotating the pathway of phagolysosome maturation 38 with the DEGs data, we noticed that, whereas DCs exposed to *Sce* and *Cal* strains showed activation (Log(FC) > 1, FDR < 0.05) of genes involved in the late phagosome maturation phase (right after the initiation of acidification of the phagosome when Rab5 is converted to Rab7), DCs exposed to *Afu* showed activation of genes relevant for the phagolysosome maturation (over-expression of cathepsins, Figure S10). Although the differences in cathepsin expression levels demand confirmation through proteomic analyses, the measurement of cytokines combined with the compliant expression of genes relevant to the pathway provided strong support for the highlighted immune response. Furthermore, cathepsins involved in the cleavage of the MHC II invariant chain 39, fundamental for antigen presentation, were also over-expressed only by DCs exposed to *Afu* (Figure S10). Similarly, the production of IL-12 and IL-23, whose genes were overexpressed after 2 hours of challenge (bottom part of Figure 3), seemed to be temporary or not translated into a functional signal, as indicated by the fact that the proteins were not significantly more abundant in the *Afu* challenge compared to other fungi (Figure 3). On the contrary, IL-1 β overexpression in response to *Afu* strains was confirmed at the protein level hours later, and hence persisted over time (Figure 3). *Cal* strains specifically induced the differential expression of 748 genes (Figure 2**B**), resulting in 8 pathways differentially represented in DC_vs*Ca* only (Figure S1). Among the pathways differentially represented only in DC_vs*Ca*, the intestinal immune network for IgA production (Figure S11), interferon alpha/beta signaling (Figure S12), and PPAR signaling pathways were of particular interest (Table S5). Genes differentially expressed in DC_vs*Cp* (Figure 2**B**) were coordinated in a well-defined module with 10 pathways differentially represented in DC_vs*Cp* only (Figure S1). Among these pathways, it is worth noting the NOD-like receptor signaling (Figure S13), MyD88:MAL(TIRAP) cascade initiated on the plasma membrane (Figure S14), and activated TAK1 mediates p38 MAPK activation pathways (Table S5). The early activation of the MyD88-MAL(TIRAP) cascade and NOD-like receptor signaling may be responsible for the late production of high levels of IL-10 and IL-23 in response to *Cpa* strains after 24 hours of challenge (Figure 3). *Sce* strains induced a largely specific response, with respectively 1820 and 23 species-specific DEGs and pathways (Figure 2**B** and Figure S1). Among the DC_vs*Sc*-specific differentially represented pathways, the interferon-gamma signaling (Figure S15) and TRAF6-mediated induction of NFkB and MAP kinases upon TLR9 activation pathways are particularly interesting (Figure S16 and Table S5). Interferon-gamma signaling was previously shown to be triggered by the recognition of *Sce* beta-glucans 40, and we found the over-expression of many of the genes with GAS promoter whose expression is regulated by IFN (Figure S15). Notably, the production of IL-12 resulting from the TLR9 activation was persistent over time, as we could quantify high levels of this cytokine even after 24 hours of DC challenge with *Sce* strains (Figure 3).

We also assessed the status of pathways known to play an important role in fungal recognition and immune response by DCs (Figure S17-S18). While genes controlled by Dectin-1 in response to fungal β -glucans were mainly over-expressed in response to *Cal* and *Sce* strains, the response mediated by DC-SIGN was activated only in response to *Sce* strains (Figure S17). Other pathways regulated by the activation of superficial receptors seemed to be variously stimulated: TLR4 in response to *Afu* and *Sce* strains, and TLR2 in response to *Cpa* strains (Figure S17). Also, endosomal receptors were involved in the immune response: TLR3 in response to *Afu*, *Cal*, and *Sce*, and TLR9 and 7 in response to *Sce* and *Cal* (Figure S18).

## DISCUSSION

**Figure 4:**
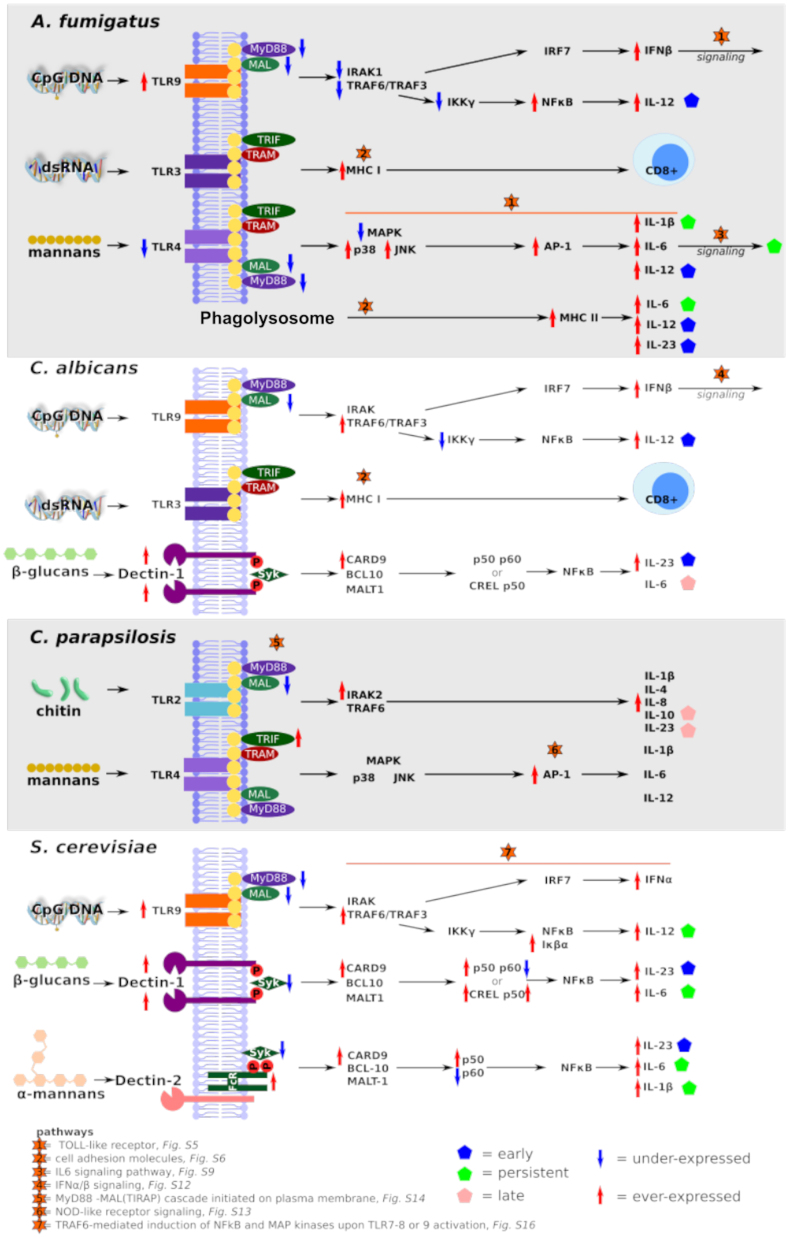
Schematic summary of the immune response triggered in DCs by the challenge with the four tested fungal species.

Transcriptional profile analysis revealed the timeline of DC intra- and intra-specific response to different fungi. Whereas differences in gene expression among different co-specific strains confirmed that the DC transcriptome effectively ‘senses’ and discriminates between strain-specific virulence factors, inter-specific comparison highlighted new insights on the immune response to fungi. *Afu* strains induced a combination of responses induced by: i- fungal DNA recognized by TLR9 as previously observed in *Cal* and *Sce* recognition 14 and resulting in an early but transient production of IL-12; ii- dsRNA recognition by TLR3, triggering MHC-I expression and potentially CD8+ differentiation 38; iii- mannans concurring to a TLR4-mediated response resulting in a transient production of IL-6 and IL-12 and persistent induction of IL-1 β ; iv- stimulation of the expression of MHC-II with following transient over-expression of IL-12 and IL-23 (Figure 4). Similarly, *Cal* strains triggered both a TLR9- and TLR3-mediated response, resulting in the transient production of IL-12. Interestingly, the interferon-alpha/beta signaling pathway, possibly activated as a consequence of TLR9 stimulation upon *Cal* recognition, was previously shown to be central for the host defense in response to *Cal* infections 39, and here we show that this response is already mounted after only 4 hours of challenge 39 (Figure 4 and Figure S12). In addition, *Cal* strains induced a Dectin-1-mediated response resulting in an early but transient production of IL-23, successively followed by a late IL-6 induction (Figure 4). *Cpa* complex has been shown to display significant differences in the cell wall compared to *Cal* with marked impacts on immune system recognition 40. Dectin-1, Mannose Receptor (MR), and TLR4 seem to be all involved in *Cpa* recognition 1, where the outermost cell wall layer, composed of *O*-linked mannans, plays a significant role in cytokine stimulation by *Cpa,* hiding the components of the inner layer, such as chitin, β 1,6- and β 1,3-glucans from recognition 41. The involvement of multiple receptors in the early recognition and response to *Candida* spp. is in line with the observation of a Dectin-1 strain-specific response 13, suggesting that some strains of this genus can mask their cell wall β -glucans and can thus escape recognition 42, hence demanding the intervention of multiple host mechanisms for the recognition, control, and eradication of the broadest intra-specific variability of the pathogen. This delay in recognition could explain the increased production of Th17-inducing IL6 in *Cal*-treated DCs (Figure 3, Figure S10).

The observed differential expression of the MyD88:MAL(TIRAP) cascade (Figure S14) suggests the involvement of TLR2 and TLR4 in the response to *Cpa* (Figure 4) as previously observed for *Cal* 43–45. This profile has been shown to occur for *Cal* 46, particularly in response to chitin (TLR2 in cooperation with dectin-1 and mannose receptor) 47 and *O*-linked mannans (TLR4) 48. TIRAP was under-expressed upon *Cpa* challenge, suggesting that, after four hours of stimulation, the immune system has already mounted a response, if considering that transcription factors and the corresponding regulated genes are overexpressed (Figure 4 and Figure S14), similarly to what previously shown for other fungal species 9. Despite these observations being gathered from transcriptomics analyses, necessitating future studies and quantification of the complex networks of proteins involved, the association of the changes in gene expression with the quantification of corresponding/signaling-related cytokines already provides robust support to the causal relationship between RNA levels and the observed immune responses. Such a response, initially resulting in the expression of IL-8 (Figure 4), later changed toward the production of IL-10 and IL-23, which are still highly induced/expressed after 24 hours upon *Cpa* challenge (Figure 3). We also observed the engagement of the cytoplasmic NOD-like receptor (NLR) signaling pathway (Figure S13). NLRs possibly cooperating in the TLR4-mediated response to *Cpa* strains have been previously shown to be involved in the response to *Candida* spp. strains through fungal cell wall component recognition 48, 49. Particularly, the mannan-activated nucleotide-binding oligomerization domain-containing protein 2 (NOD2) was previously shown to be involved in *Cpa* recognition, inducing a strong downstream cytokine response 49, 50.

It has been previously suggested that TLRs and NLRs cooperate in mounting a species-, strain- and morphotype-specific response, defining the final induced adaptive response (Th1/Th2/Th17) 51, 52. *Sce* strains, together with *Afu* strains, induced the most complex response, resulting in a combination of several different immune pathways: i- the activation of Dectin-1, potentially via recognition of β -glucans 52, resulting in an early production of IL-23 and persistent production of IL-6; ii - the activation of Dectin-2 resulting in a persistent production of IL-6 and IL-1 β , and iii - a persistent activation of the endosomal TLR9 receptor, resulting in the production of IL-12 and IL-10 48 and which trafficking to the endosomes is driven by both β -glucans recognition 53 and chitin 48 (Figure 4). To note, whereas the IL-6 gene was over-expressed in DC_vs*Af* after 4 hours of challenge, the genes involved in the IL6 signaling pathway were mostly under-expressed, besides the gene *SOCS3* (Suppressor Of Cytokine Signaling 3), known to inhibit cytokine production through a feedback mechanism 54. Furthermore, after 24 hours, the amount of IL-6 produced by DCs was lower in response to the specific species compared to *Sce* and *Cal*. IL-6 is a potent regulator of DC functions 55, indicating that differences in the activation timing of the IL-6 signaling pathway among various yeast species are crucial for studying species-specific immune responses. At the same time, it has been proven that the loss of activation of the IL-6 signaling pathway results in an impaired activity of phagolysosomes 37. Annotating the pathway of phagolysosome maturation 38 with the DEGs data (Figure S10), we noticed that, whereas DCs exposed to *Sce* and *Cal* strains showed activation (Log(FC) > 1, FDR < 0.05) of genes involved in the late phagosome maturation phase (right after the initiation of acidification of the phagosome, when Rab5 is converted to Rab7), DCs exposed to *Afu* showed activation of genes relevant for the phagolysosome maturation (over-expression of cathepsins, Figure S10). Furthermore, cathepsins involved in the cleavage of the MHC II invariant chain 39, fundamental for antigen presentation, were also overexpressed only by DCs exposed to *Afu* (Figure S10). The observation, based on DEGs analysis, about the IL-6 signaling pathway and phagolysosome activation led us to hypothesize that the response of DCs to *Aspergillus fumigatus* conidia is characterized by an early activation followed by an over-expression of MHC-II (Figure S6). Accordingly, our previous study associated the observation that different fungal species survived differently to the acidification of the phagolysosome with a shift between Th1 and Th17 responses 14. Despite the complexity of such interconnections and the inability to detect some specific Pattern Recognition Receptors (PRRs) engagements, such as the ones from MR, our study allowed us to capture the interplays occurring among different PRRs signaling in mounting the appropriate and complex responses to the diverse fungal infection. Our results indicate that while the response to *Sce* and *Cal* seems to be mainly driven by β -glucans and fungal DNA, with *Sce* also recognized through mannan recognizing receptor, gene expression signaling towards *Afu* challenge seems to be mainly supported initially by mannans and chitin, and later by fungal DNA recognition. This is in agreement with a more severe early inflammatory response elicited by *Afu* compared to *Cal* and *Sce*, suggesting that the role of commensals and opportunistic pathogens of these two yeasts is nicely recapitulated in their transcriptional profile.

The transcriptomic profiling of DCs exposed to four pathogenic and non-pathogenic fungal species confirmed both a common core and a species-specific immune response to fungi 11–15. Transcriptional profiles highlighted the signaling pathways specifically activated in response to different fungal species. Other biochemical assays (e.g., qRT-PCR, proteins, or cytokines/chemokines quantification), which should be focused on specific and pre-selected targets, would not be able to provide a level of information of this depth.

Intriguingly, we were able to identify a time-dependent core response mounted by DCs in response to both pathogenic and non-pathogenic fungi. Previous studies have shown that strains belonging to non-pathogenic species may induce an inflammatory immune response 16. Together, these findings suggest that co-specific non-pathogenic fungal strains can trigger an inflammatory or tolerogenic response. Concordantly, the GRAS yeast *Sce* widely affected the transcriptional status of DCs, inducing a greater modulation in gene expression than the pathogenic species *Cal* and *Cpa*; this results suggest that the ability to hide to the immune system is one of the main traits for pathogenicity and again indicating that the early activation in gene expression of markers of inflammation or pathogenicity should be seen as a distinctive trait for commensals. As a whole, our study highlights how transcriptomics can help obtain a complete portrait of the immune response to pathogenic and commensal fungal species and how this picture is strongly influenced by strain-specific traits, including its origin. Such analyses represent a powerful approach to a strain-level investigation of the pathogenic potential of yeasts, using the dendritic cell transcriptome as a readout.

## MATERIALS AND METHODS

### Human monocyte-derived dendritic cell preparation and challenges

Dendritic cells (DCs) were differentiated from human Peripheral blood mononuclear cells (PBMCs)-isolated monocytes, and cultured in complete RPMI medium, 37 ${}^{\circ }$ C, 5% CO ${}_{2}$ . PBMCs were collected from buffy coat blood samples from 3 healthy donors from the Transfusion Unit of the Careggi Hospital (Florence, Italy) by Ficoll-Hypaque density gradient centrifugation (Biochrom AG). Monocytes were isolated from low-density PBMCs by magnetic enrichment with anti-CD14 beads (Miltenyi Biotec). Cells were cultured in RPMI complete medium (RPMI 1640, 10% w/v FBS, 1% w/v 200 mM glutamine, 1% w/v 50 mM sodium pyruvate, penicillin-streptomycin) in the presence of GM-CSF (800 U/ml) and recombinant IL-4 (1000 U/ml) for 6 days to allow dendritic cell (DC) differentiation (37 ${}^{\circ }$ C, 5% CO ${}_{2}$ ). DCs were cultured for 4 hours with the different live fungal strains at a stimulus:DC ratio of 5:1 (37 ${}^{\circ }$ C, 5% CO ${}_{2}$ , complete RPMI medium) for RNA extraction or 24 hours with 2 h UV-treated fungal preparations for cytokines quantification. As controls, DCs were grown alone in the same experimental conditions.

### Fungal cell preparation

Five *S. cerevisiae* strains, listed in Table S1, were grown at 28 ${}^{\circ }$ C with stirring (150 r.p.m.) until mid-exponential phase in liquid YPD medium (1% w/v yeast extract, 2% w/v peptone, and 2% w/v glucose). Cells were then harvested by centrifugation, washed twice with sterile water, and resuspended in fresh RPMI 1640 complete medium.

Three *C. albicans* strains (the reference strain SC5314, and 2 mistranslated strains) and 2 *C. parapsilosis* strains (YA4 and YB1) 12 were grown at 28 ${}^{\circ }$ C with stirring (150 r.p.m.) until mid-exponential phase in liquid Sabouraud medium (1%, w/v peptone and 2% w/v glucose). Cells were harvested as indicated above.

Three *A. fumigatus* strains, namely AF293 and its color mutants AF293, AF293 VKBR1, and AF293 VKWH2 9, were grown at 37 ${}^{\circ }$ C in Sabouraud agar medium. Abundant conidia were produced under these conditions. Conidia were harvested by gently scraping the slant’s surface in PBS + 0.1% Tween and washed twice with sterile water. To remove hyphae and debris, the conidial suspension was filtered through four layers of sterile gauze and resuspended in fresh complete RPMI 1640.

For fungal challenges, the *Sce, Cal, Cpa,* and *Afu* strains pre-cultured as described were added to DCs to a final fungus:DC ratio of 5:1.

### Quantification of cytokines

The pro-inflammatory cytokines IL-6, IL-23, IL-12p70, IL-1 β , and TNF α were quantified in 24-hour supernatants from DCs challenged with the different fungal preparations or untreated (control) using the Milliplex® MAP human cytokine/chemokine kit (Millipore), according to the manufacturer’s instructions.

### RNA extraction, RNA-seq library preparation, and sequencing

Dendritic cells from three healthy human donors were cultured in the presence or absence of *Aspergillus fumigatus*, *Saccharomyces cerevisiae*, *Candida albicans,* or *C. parapsilosis* strains. After 4 hours of incubation, total RNA was extracted from each sample. RNA sequencing was used to evaluate differences in the transcriptomes of human cells challenged and unchallenged with each fungal species. FASTQ files and BAM files with alignments and analysis results have been deposited in ArrayExpress Archive under accession E-SYBR-2 (https://www.ebi.ac.uk/arrayexpress/experiments/E-SYBR-2/). Total RNA was extracted from cells using the standard TriZol protocol, according to the manufacturer’s instructions. For *A. fumigatus*-containing samples, before TriZol extraction, fungal cells were disrupted by grinding in liquid nitrogen with a pestle and mortar. Extracted RNAs were then quantified, and their integrity was checked using NanoChip BioAnalyzer technology (Agilent). For each sample, one paired-end mRNA library with approximately 150 bp insert size was prepared using the TruSeq RNA Sample Preparation Kit v2 (Illumina). Libraries were sequenced on Illumina HiSeq2000 lanes using 2x75 bp reads. The cluster generation kit was TruSeq PE Cluster Kit v3-cBot-HS, and the sequencing kit was TruSeq SBS Kit v3-HS. More than 30 million reads were generated for each sample. Sequencing reads were deposited in ArrayExpress Archive under accession E-SYBR-2 (Table S1).

### RNA-sequencing data analysis

RNA-seq data analysis was performed on 41 samples from three healthy human donors and 13 fungal strains (Table S1). A quality check was carried out using FastQC (http://www.bioinformatics.babraham.ac.uk/), followed by manual checking.

### Differential expression analysis

Reads were aligned to the GRCh38.12 human genome assembly (Ensembl) with tophat2 56, and transcript abundances were quantified using HTSeq-count 57. Raw counts were filtered to exclude genes that did not have at least 2 counts per million. Differential expression analysis was conducted using edgeR v2.99 58, computing for each gene the logFC, calculated as the logarithm to base 2 of the number of reads in DCs challenged with the fungus versus the number of reads in unchallenged DCs (fold change). The significance of differential expression was tested through the quasi-likelihood F-test test corrected with the Benjamini and Hochberg false discovery rate method 59. A gene was considered differentially expressed (DEG) when showing FDR < 0.05.

### Pathway analysis

Genes found to be differentially expressed were further investigated using pathway and gene ontology enrichment analysis. Pathway enrichment analysis was carried out using David 60, KEGG (Kyoto Encyclopedia of Genes and Genomes) 61, and Reactome (https://reactome.org/download-data/, accessed December 2018) knowledge databases.

## ETHICS APPROVAL

The experimental plan was approved by the local Ethical Committee of Azienda Universitaria Ospedaliera Careggi (AUOC, Careggi Hospital, Florence, Italy), and written informed consent was obtained from all donors (approval document n. 87/10). The study was designed in conformity with the international recommendation (Dir. EU 2001/20/EC) and its Italian counterpart (DM 15 Luglio 1997; D.Lvo 211/2003; D.L.vo 200/2007) for clinical trials and following the Declaration of Helsinki, to assure protection and care of subjects involved.

## SUPPLEMENTAL MATERIAL

All supplemental data for this article are available online at http://microbialcell.com/researcharticles/2026a-rizzetto-microbial-cell/.

Click here for supplemental data file.

Click here for supplemental data file.

Click here for supplemental data file.

Click here for supplemental data file.

Click here for supplemental data file.

Click here for supplemental data file.

## CONFLICT OF INTEREST

The authors declare no competing interests.

## ABBREVIATIONS

DCs – dendritic cells

DEG – differentially expressed gene

GRAS – Generally Recognized As Safe

MDS – MultiDimensional Scaling

MR – mannose receptor

NLR – NOD-like receptor

PBMC – -peripheral blood mononuclear cells

PRR – Pattern Recognition Receptors
