# Supplementary material for: The transcriptome of dendritic cells redraws the boundaries between pathogenicity and commensalism in yeast
Source: Microb Cell. 2026 Jul 7;13:282–92. doi: 10.15698/mic2026.07.882 (PMC13365740; doi:10.15698/mic2026.07.882)

Supplementary Information

For

Transcriptomes of dendritic cells encountering commensal and pathogenic fungi highlight different response mechanisms and timing

Lisa Rizzetto, Irene Stefanini, Stefano Nenciarini, Samantha Riccadonna, Ivo G. Gut, Marta Gut, Simon Heath, Duccio Cavalieri

This pdf file includes:

Supplementary Figures

Figure S1: Venn diagrams comparing GOs and pathways differentially represented in DC treated with the tested fungal species.

Figure S2: Expression levels of genes involved in the cytokine-cytokine receptor interaction pathway.

Figure S3: TNF signaling pathway and expression of the involved genes.

Figure S4: MAPK signaling pathway and expression of the involved genes.

Figure S5: TOLL_like receptor signaling pathway and expression of the involved genes.

Figure S6: Cell adhesion molecules pathway and expression of the involved genes.

Figure S7: Inflammatory bowel disease pathway and expression of the involved genes.

Figure S8: Chemokine signaling pathway and expression of the involved genes.

Figure S9: Heatmap of genes involved in IL-6 signaling pathway.

Figure S10: Quantification of cytokines produced by DCs challenged with strains of four fungal species.

Figure S11: Intestinal immune network for IgA production pathway and expression of the involved genes.

Figure S12: Heatmap of genes involved in IFN alpha/beta signaling.

Figure S13: NOD-like receptor signaling pathway and expression of the involved genes.

Figure S14: MyD88-MAL(TIRAP) cascade initiated on plasma membrane pathway and expression of the involved genes.

Figure S15: Interferon gamma signaling pathway and expression of the involved genes.

Figure S16: TRAF6 mediated induction of NFkB and MAP kinases upon TLR7-8 or 9 activation pathway and expression of the involved genes.

Figure S17: Known pathways involved in the DCs antifungal response to fungi triggered by surface receptors.

Figure S18: Known pathways involved in the DCs antifungal response to fungi triggered by endosomal receptors.

Supplementary Tables

Table S1: Fungal strains used in this study and references to sequence data deposited in ArrayExpress Archive.

Table S2 ("Table2_DEGs_GOs_pathways.xls"): Transcriptional profiles (DEGs, Gos, and pathways) of dendritic cells (DCs) exposed to four fungal species compared to unchallenged DCs.

Table S3 ("Table3_shared_DEGs_GOs_pathways.xls"): Common features among transcriptional profiles of DCs challenged with *A. fumigatus*, *C. albicans*, *C. parapsilosis*, and *S. cerevisiae*.

Table S4 ("Table4_species-specific_DEGs_GOs_pathways.xls"): Features characterizing transcriptional profiles of DCs challenged with *A. fumigatus*, *C. albicans*, *C. parapsilosis*, or *S. cerevisiae*.

Figures

Figure S1

Venn diagrams comparing pathways differentially represented in DC treated with the tested fungal species. Differentially represented pathways were identified for every challenge, then the resulting lists were compared. Differentially represented pathways are listed in **Table S2**, the lists of shared and species specific pathways are in **Table S3** and **S4** respectively.

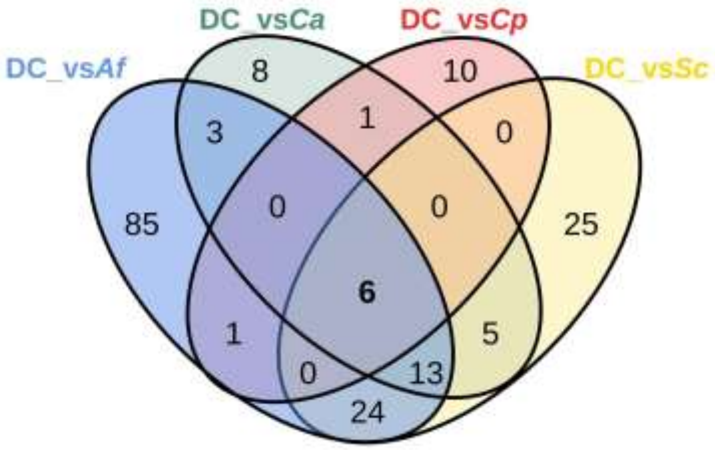

Figure S2

Expression levels of genes involved in the cytokine-cytokine receptor interaction pathway. This pathway was found to be differentially represented in DCs treated with any tested fungal species. Pathway enrichment analysis was carried out with David (Huang et al. 2009); the pathway map was obtained from the KEGG database (Kanehisa et al. 2017) and drawn with pathview (Luo et al. 2017).

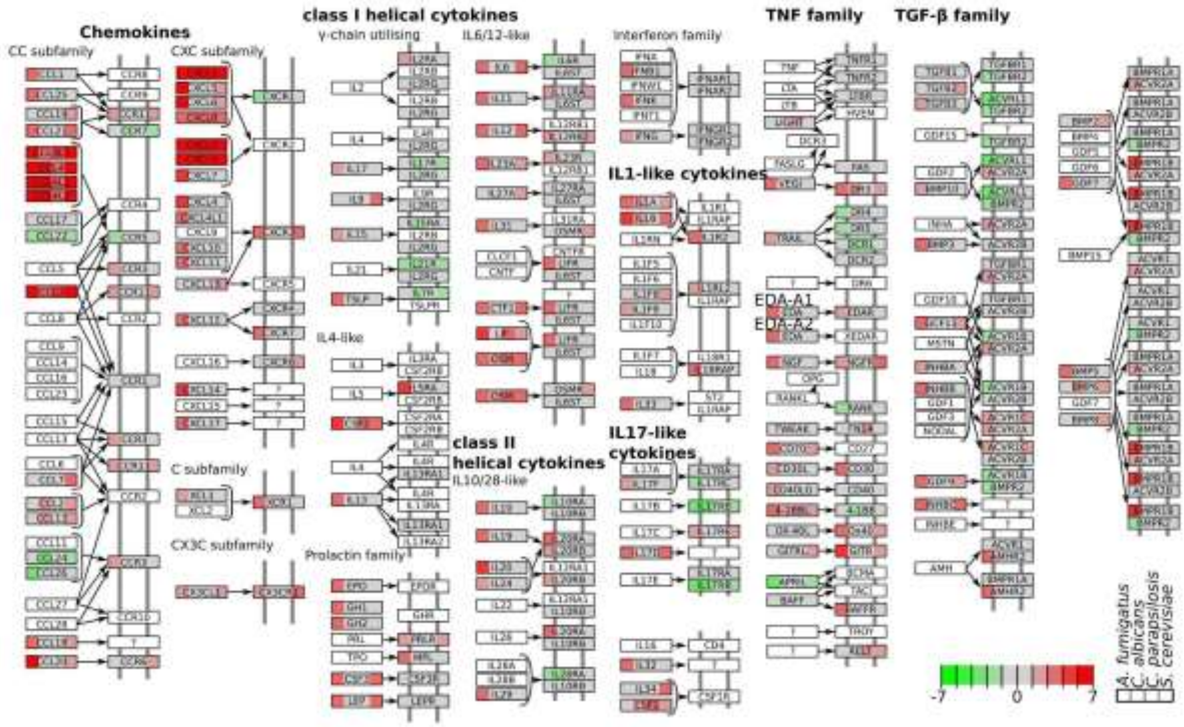

Figure S3

TNF signaling pathway and expression of the involved genes. This pathway was found to be differentially represented in DCs treated with any tested fungal species. Pathway enrichment analysis was carried out with David (Huanget al. 2009); the pathway map was obtained from the KEGG database (Kanehisa et al. 2017) and drawn with pathview (Luo et al. 2017).

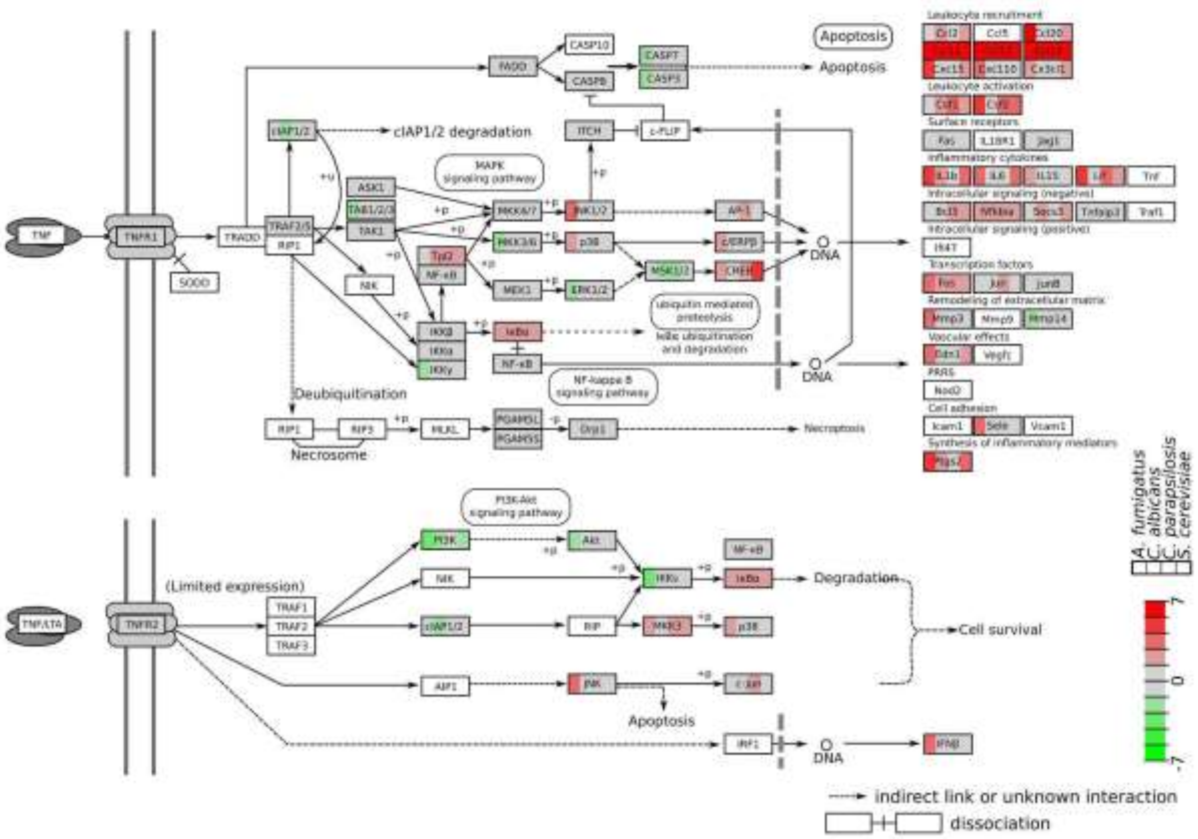

Figure S4:

MAPK-signaling pathway and expression of the involved genes. This pathway was found to be differentially represented in DCs treated with any tested fungal species. Pathway enrichment analysis was carried out with David (Huanget al. 2009); the pathway map was obtained from the KEGG database (Kanehisa et al. 2017) and drawn with pathview (Luo et al. 2017).

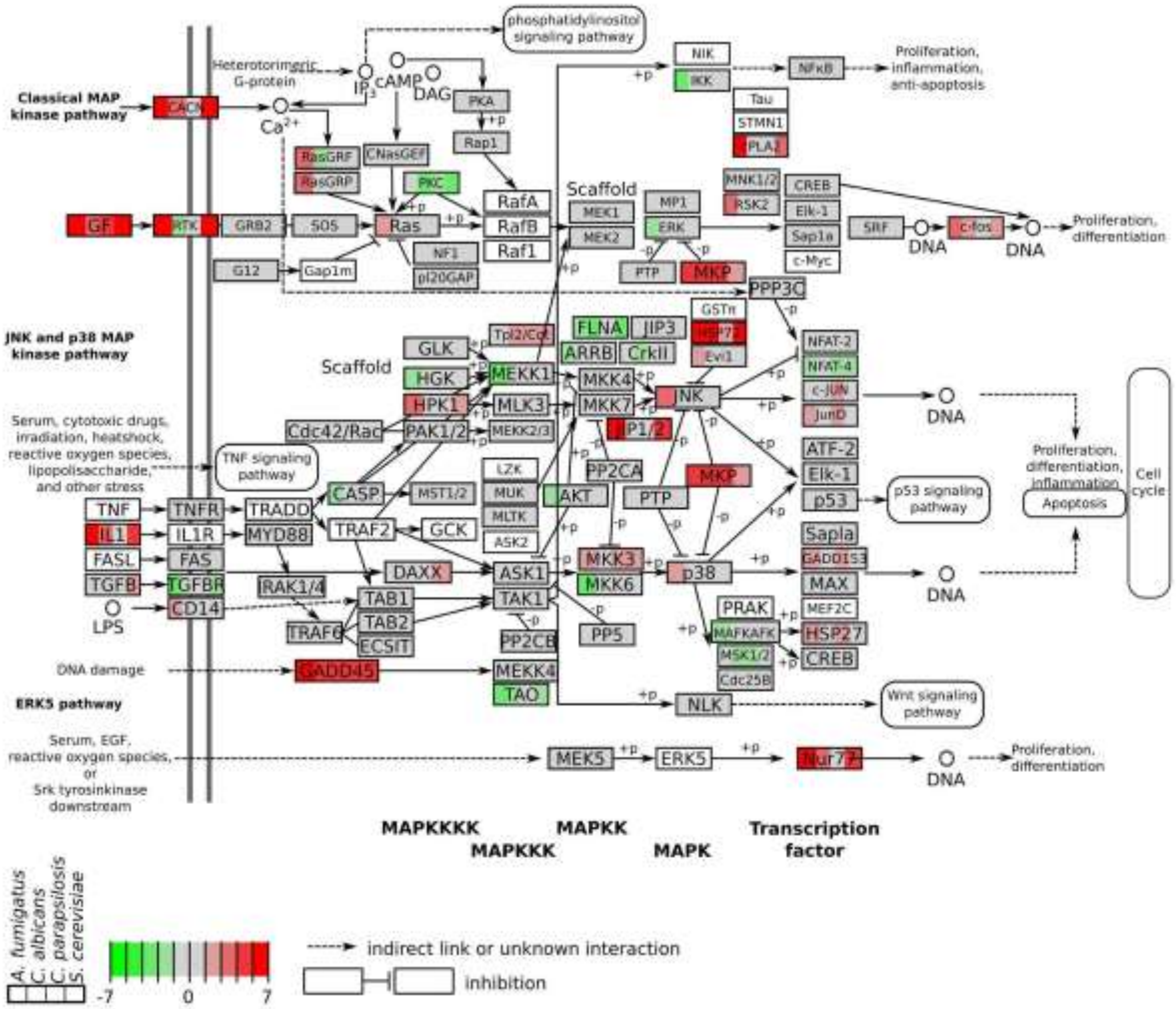

Figure S5

TOLL-like receptor signaling pathway and expression of the involved genes. This pathway was found to be differentially represented in DCs treated with *A. fumigatus* and *C. parapsilosis* strains. Pathway enrichment analysis was carried out with David (Huanget al. 2009); the pathway map was obtained from the KEGG database (Kanehisa et al. 2017) and drawn with pathview (Luo et al. 2017).

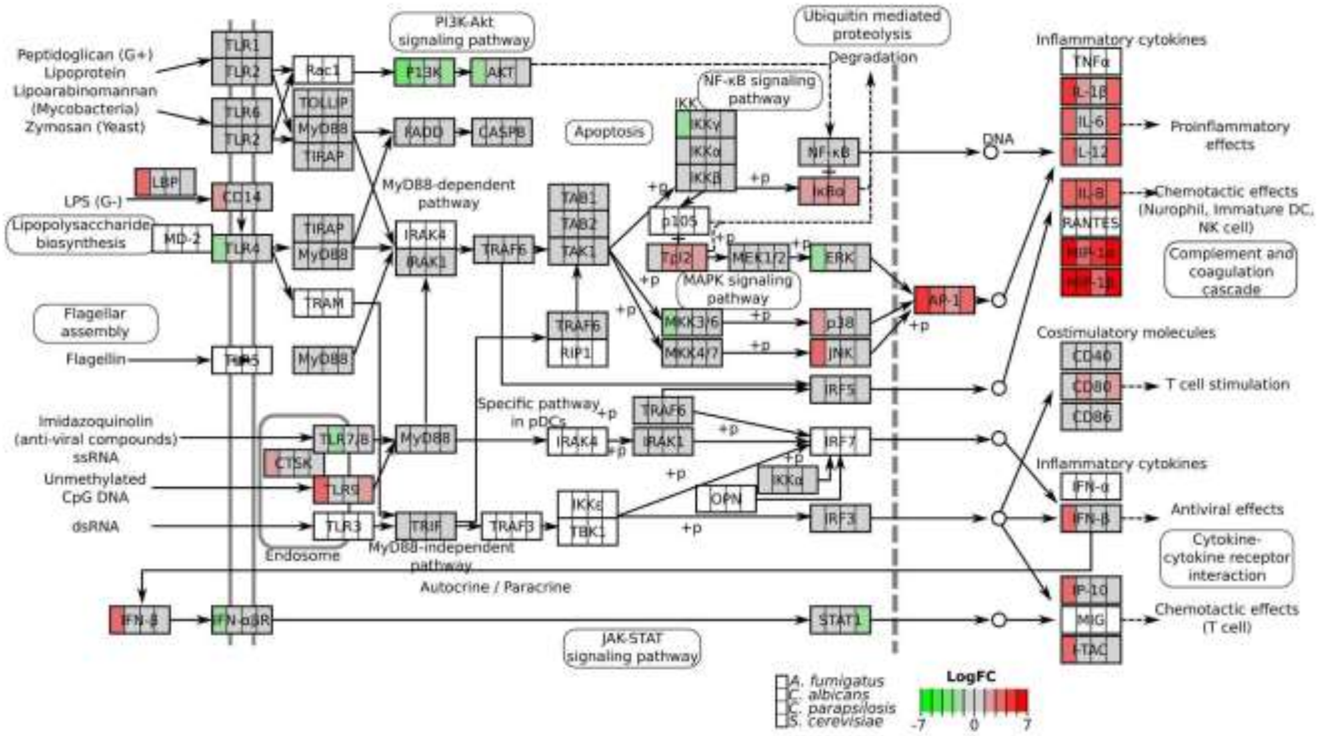

Figure S6

Cell adhesion molecules pathway and expression of the involved genes. This pathway was found to be differentially represented in DCs treated with *A. fumigatus* and *C. albicans* strains. Pathway enrichment analysis was carried out with David (Huanget al. 2009); the pathway map was obtained from the KEGG database (Kanehisa et al. 2017) and drawn with pathview (Luo et al. 2017).

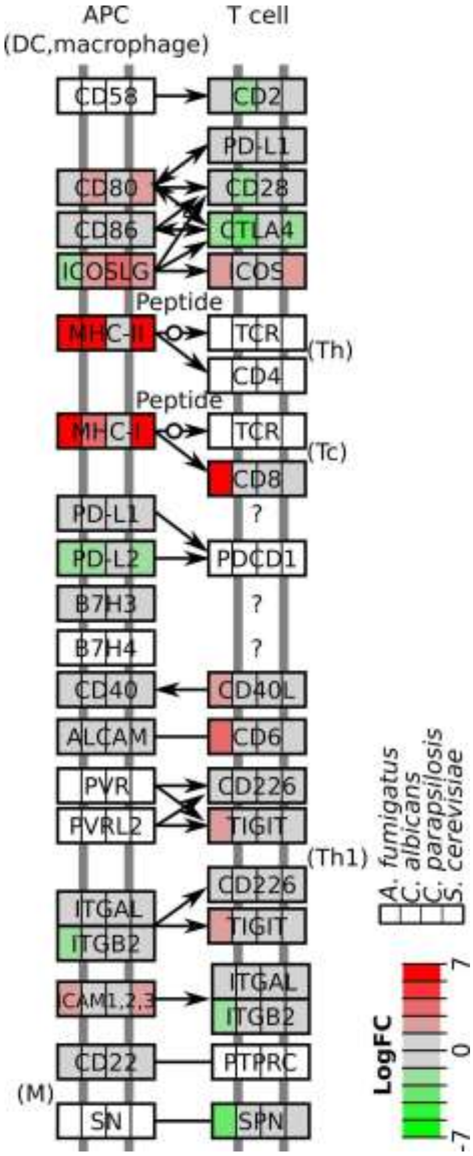

Figure S7:

Inflammatory bowel disease pathway and expression of the involved genes. This pathway was found to be differentially represented in DCs challenged with *A. fumigatus* and *S. cerevisiae* strains. Pathway enrichment analysis was carried out with David (Huanget al. 2009); the pathway map was obtained from the KEGG database (Kanehisa et al. 2017) and drawn with pathview (Luo et al. 2017).

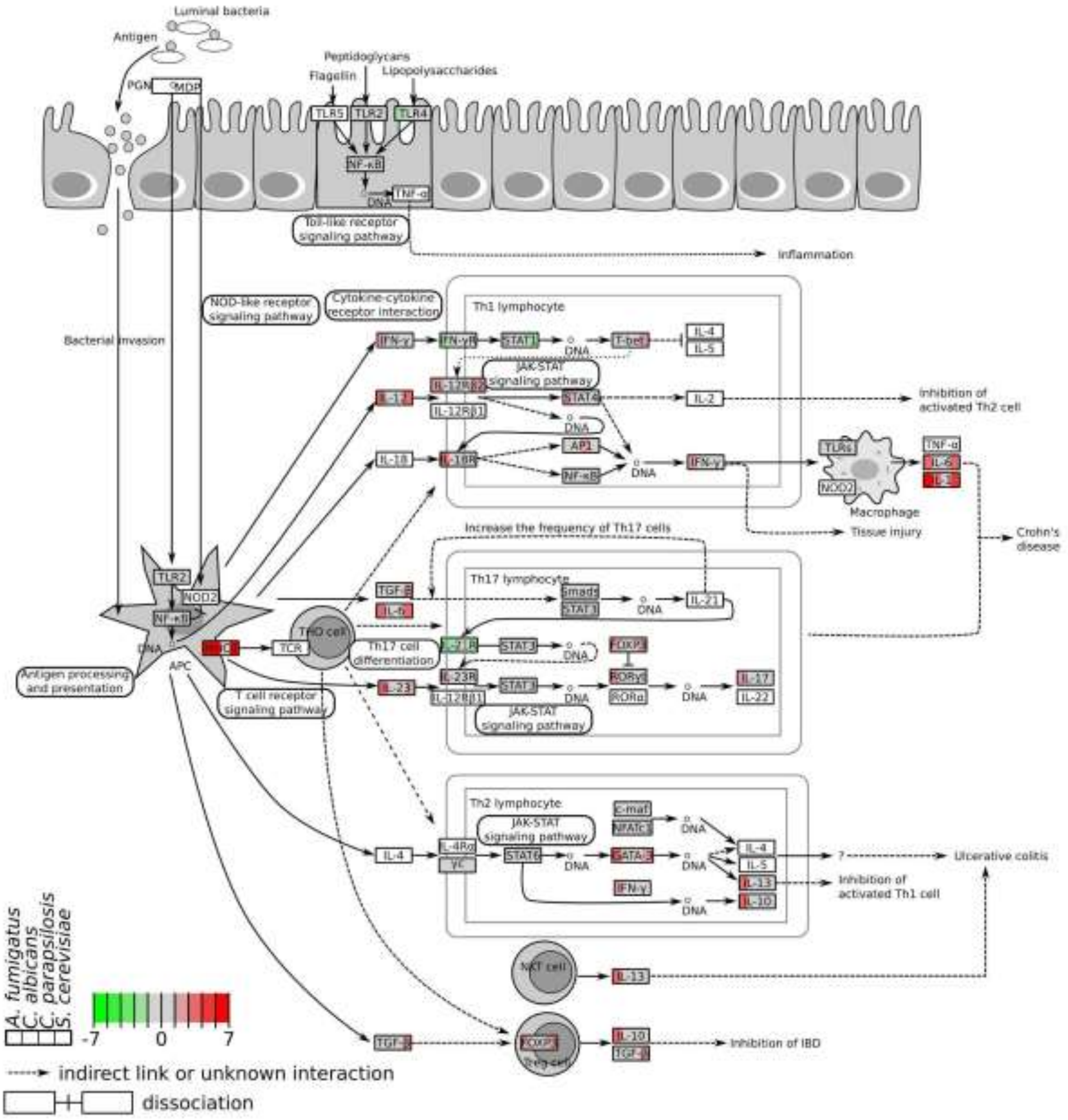

Figure S8

Chemokine signaling pathway and expression of the involved genes. This pathway was found to be differentially represented in DCs challenged with *A. fumigatus* strains. Pathway enrichment analysis was carried out with David (Huanget al. 2009); the pathway map was obtained from the KEGG database (Kanehisa et al. 2017) and drawn with pathview (Luo et al. 2017).

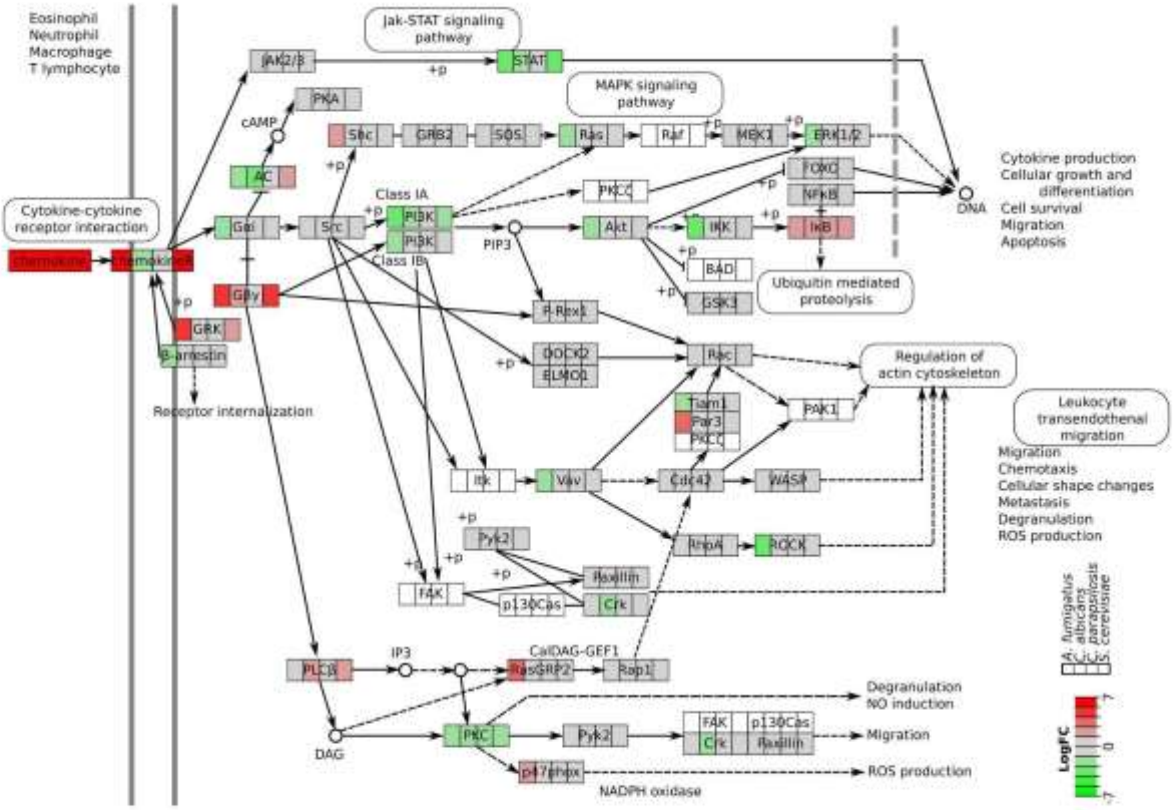

Figure S9

Heatmap of genes involved in IL-6 signaling pathway.

The IL-6 signaling pathway was found to be differentially represented in DC_vs*Af* (Dendritic Cells exposed to *A. fumigatus* strains). The heatmap shows the genes belonging to this pathway and differentially represented in DC_vs*Af*.

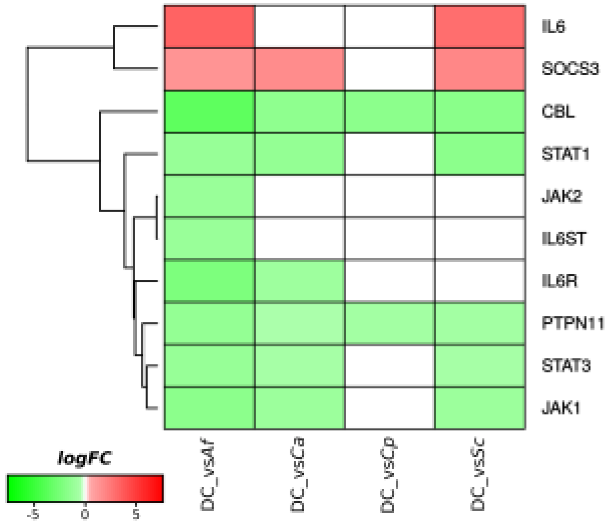

Figure S10

Phagolysosome maturation pathway and expression of the involved genes. The pathway was drawn according to Pauwels et al. 2017, and, for each step involving known genes, it was annotated with the DCs DEGs data gathered over this study. *=DEGs with Log(FC)>1, FDR<0.05. #: to have an impact on v-ATPase functions, the expression of coupled V0 and V1 moieties has to be concerted, hence, the observation of the over- or under-expression of these genes does not provide information on the functional consequences.

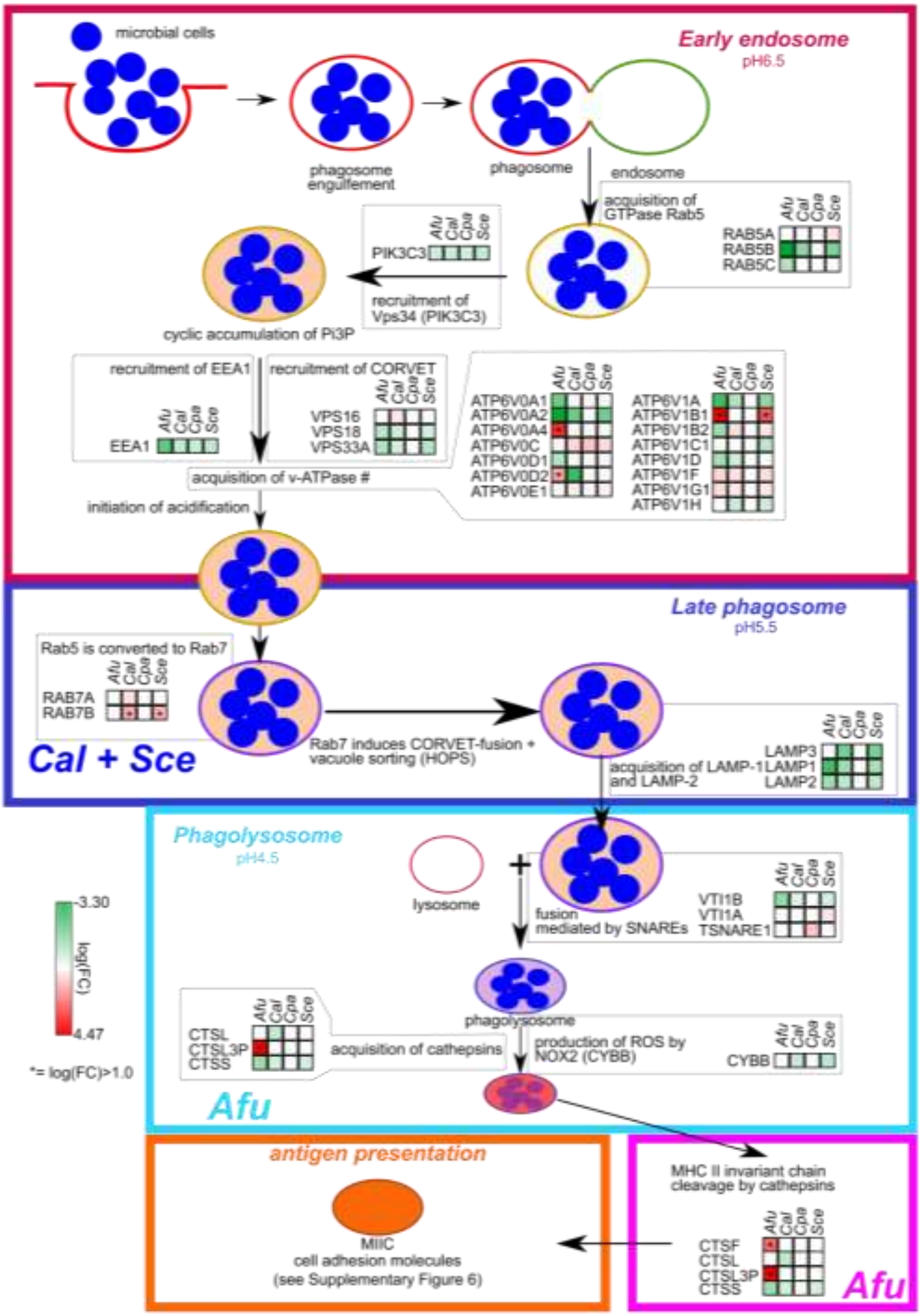

[Figure S11](#)

Intestinal immune network for IgA production pathway and expression of the involved genes. This pathway was found to be differentially represented in DCs challenged with *C. albicans* strains. Pathway enrichment analysis was carried out with David (Huanget al. 2009); the pathway map was obtained from the KEGG database (Kanehisa et al. 2017) and drawn with pathview (Luo et al. 2017).

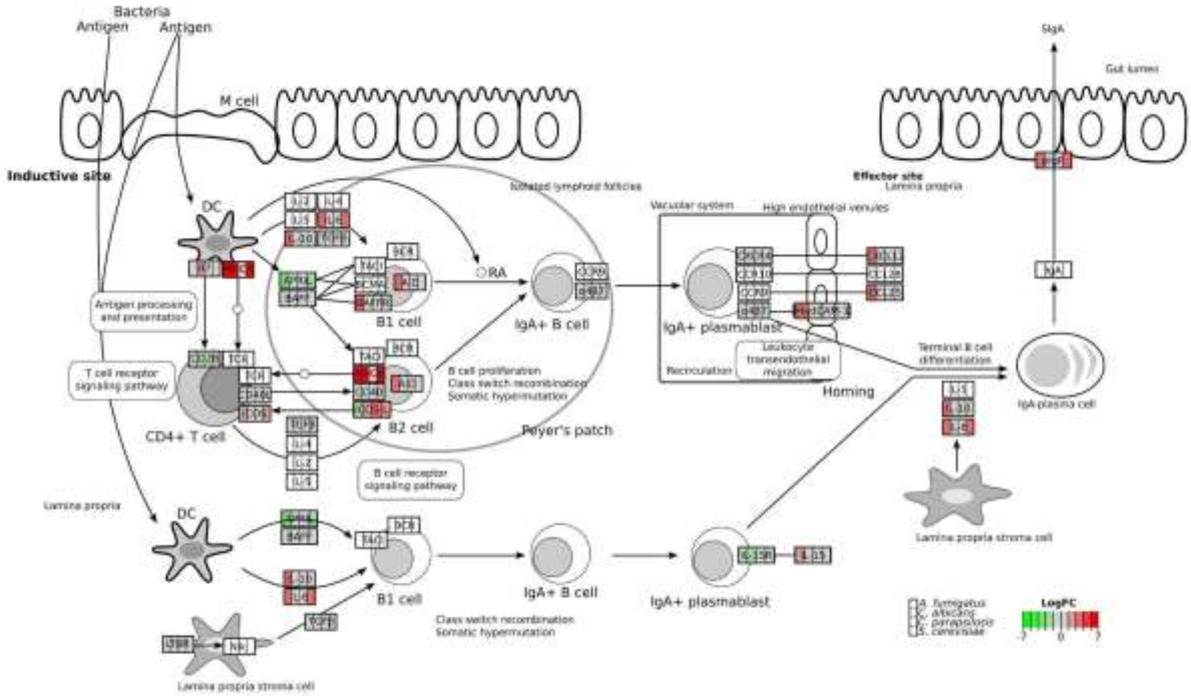

Figure S12

Heatmap of genes involved in IFN alpha/beta signaling.

The IFN alpha/beta signaling pathways was found to be differentially represented in DC_vs*Ca* (Dendritic Cells exposed to *C. albicans* strains).

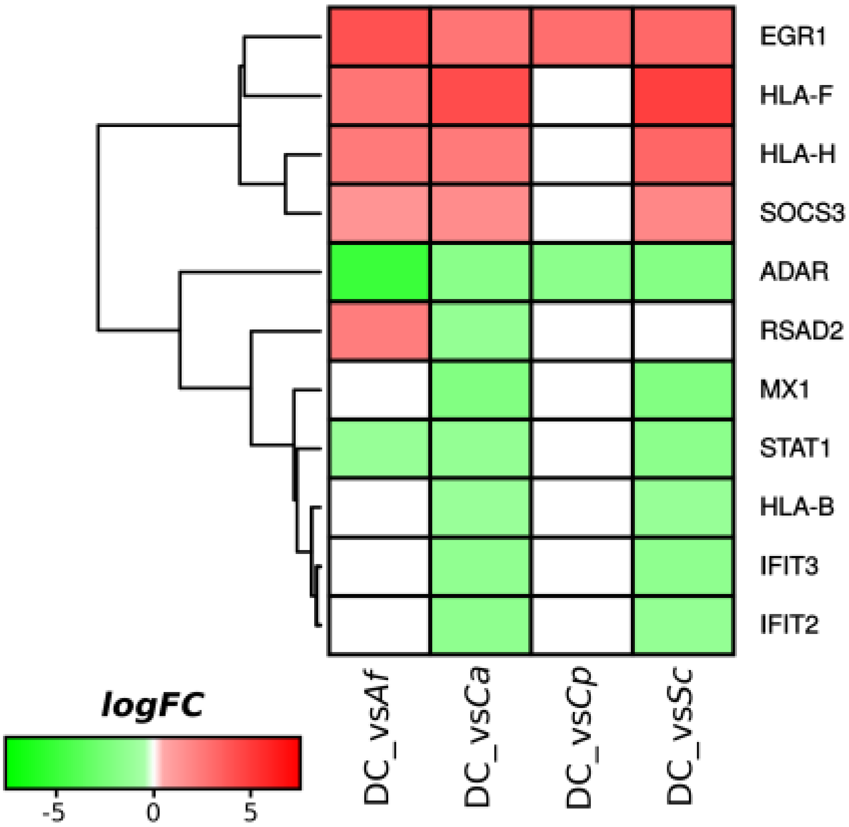

Figure S13

NOD-like receptor signaling pathway and expression of the involved genes. This pathway was found to be differentially represented in DCs challenged with *C. parapsilosis* strains. Pathway enrichment analysis was carried out with David (Huanget al. 2009); the pathway map was obtained from the KEGG database (Kanehisa et al. 2017) and drawn with pathview (Luo et al. 2017).

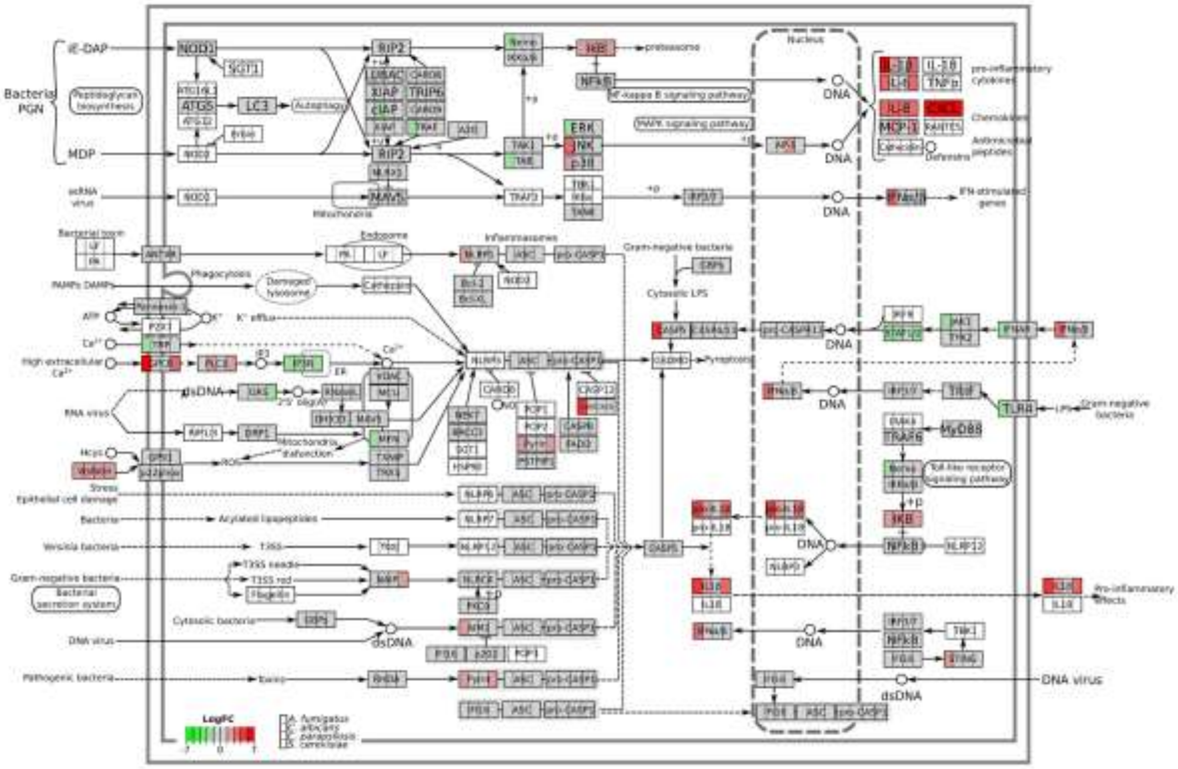

Figure S14

MyD88-MAL(TIRAP) cascade initiated on plasma membrane. This pathway was found to be differentially represented in DCs challenged with *C. parapsilosis* strains. Pathway enrichment analysis was carried out with David (Huanget al. 2009); the pathway map was obtained from the KEGG database (Kanehisa et al. 2017) and drawn with pathview (Luo et al. 2017).

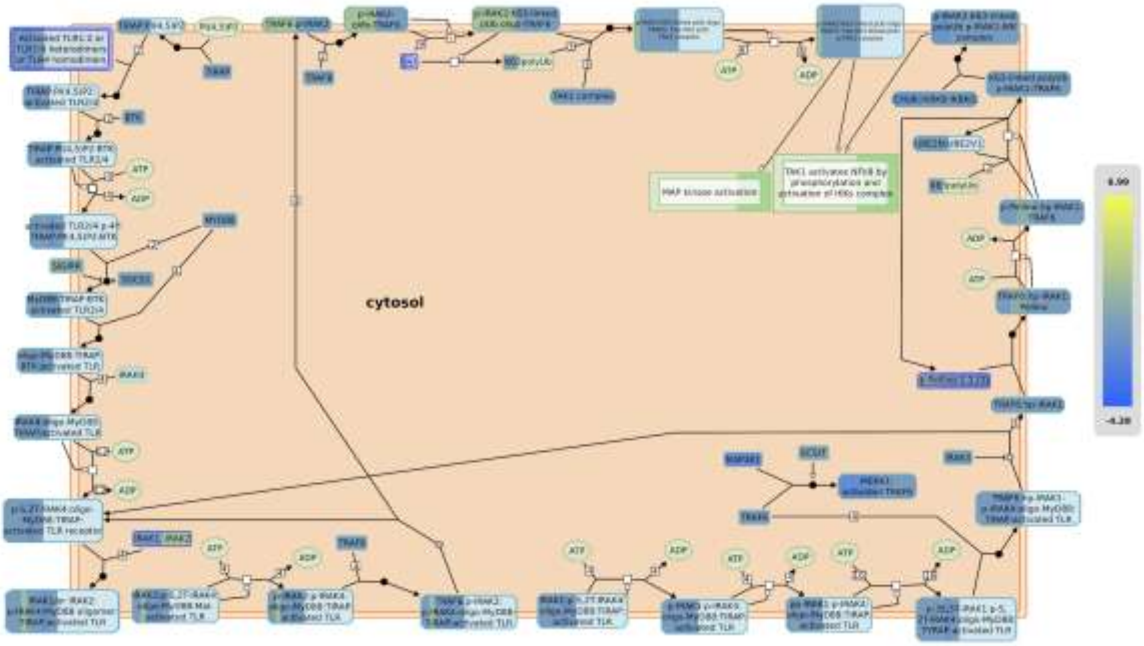

Figure S15

Interferon gamma signaling pathway and expression of the involved genes. This pathway was found to be differentially represented in DCs challenged with *S. cerevisiae* strains. Pathway enrichment analysis was carried out with David (Huanget al. 2009); the pathway map was obtained from the KEGG database (Kanehisa et al. 2017) and drawn with pathview (Luo et al. 2017).

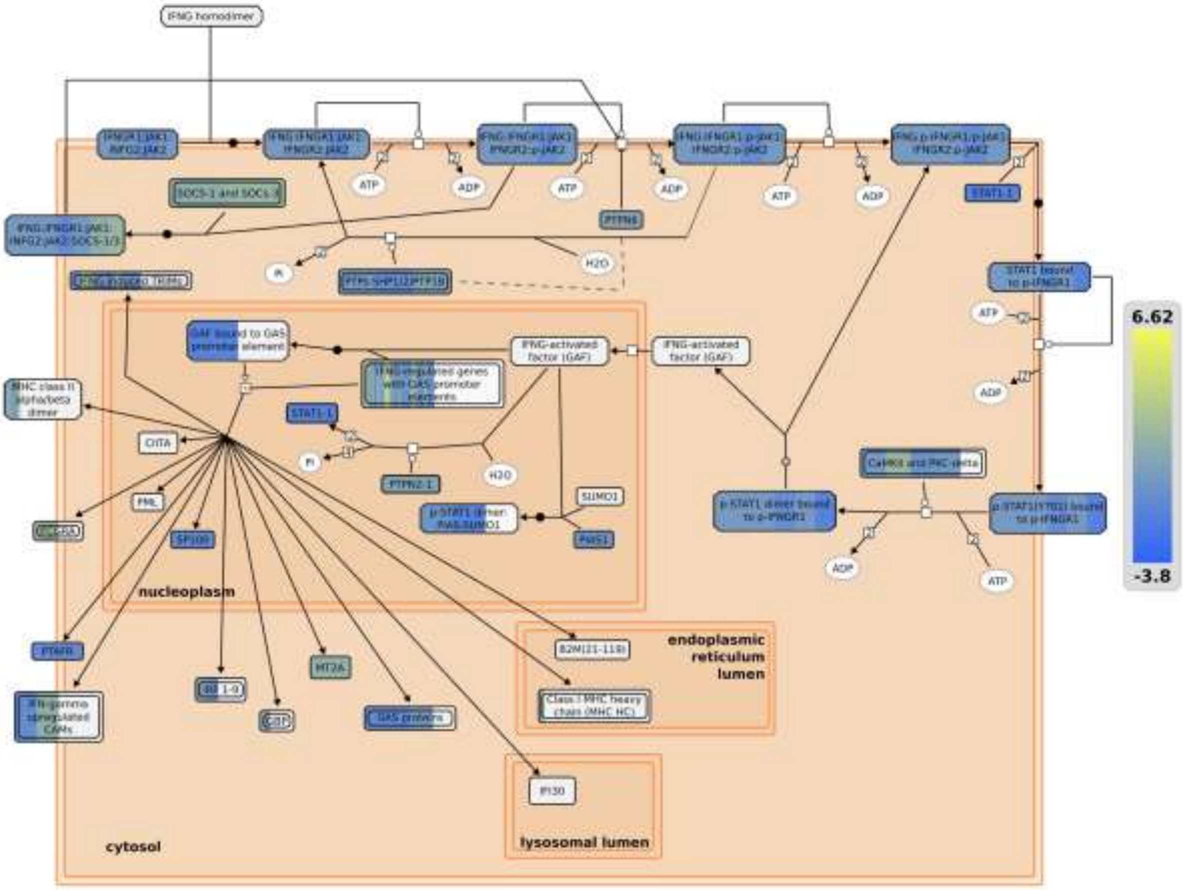

Figure S16

TRAF6 mediated induction of NFkB and MAP kinases upon TLR7-8 or 9 activation pathway and expression of the involved genes. This pathway was found to be differentially represented in DCs challenged with *S. cerevisiae* strains. Pathway enrichment analysis was carried out with David (Huanget al. 2009); the pathway map was obtained from the KEGG database (Kanehisa et al. 2017) and drawn with pathview (Luo et al. 2017).

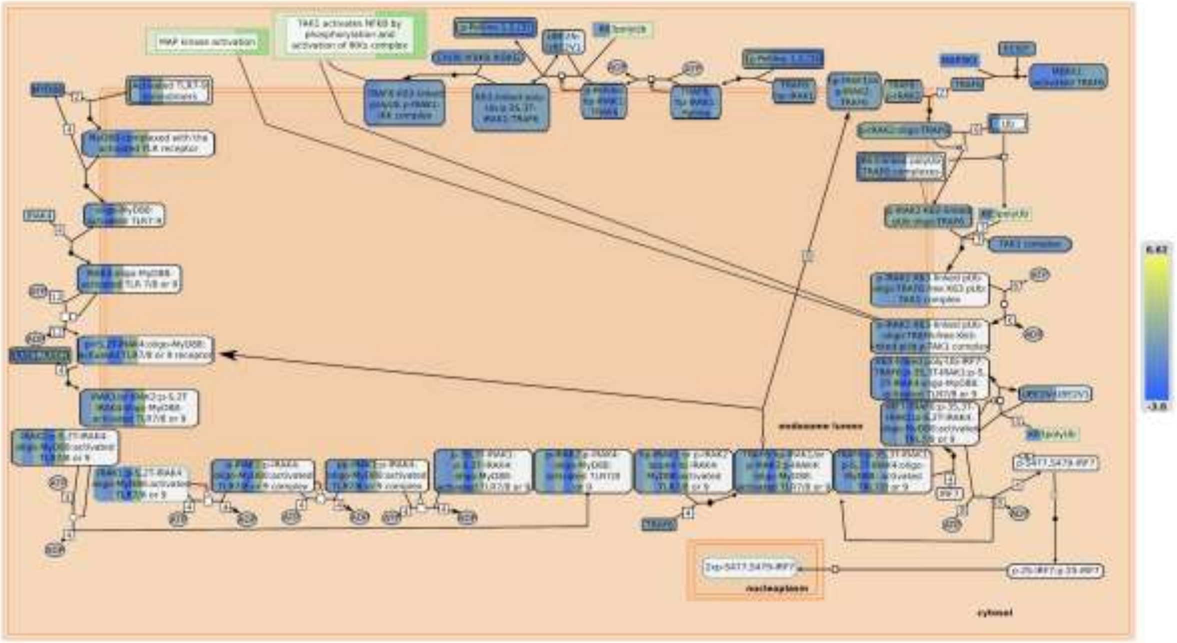

Figure S17

Known pathways involved in the DCs antifungal response to fungi triggered by surface receptors. The figure has been inspired by the schemes proposed by Ramirez-Ortiz and Means (Virulence 2012, 3:1-12) and Romani (Nature 2011, 11:275-288).

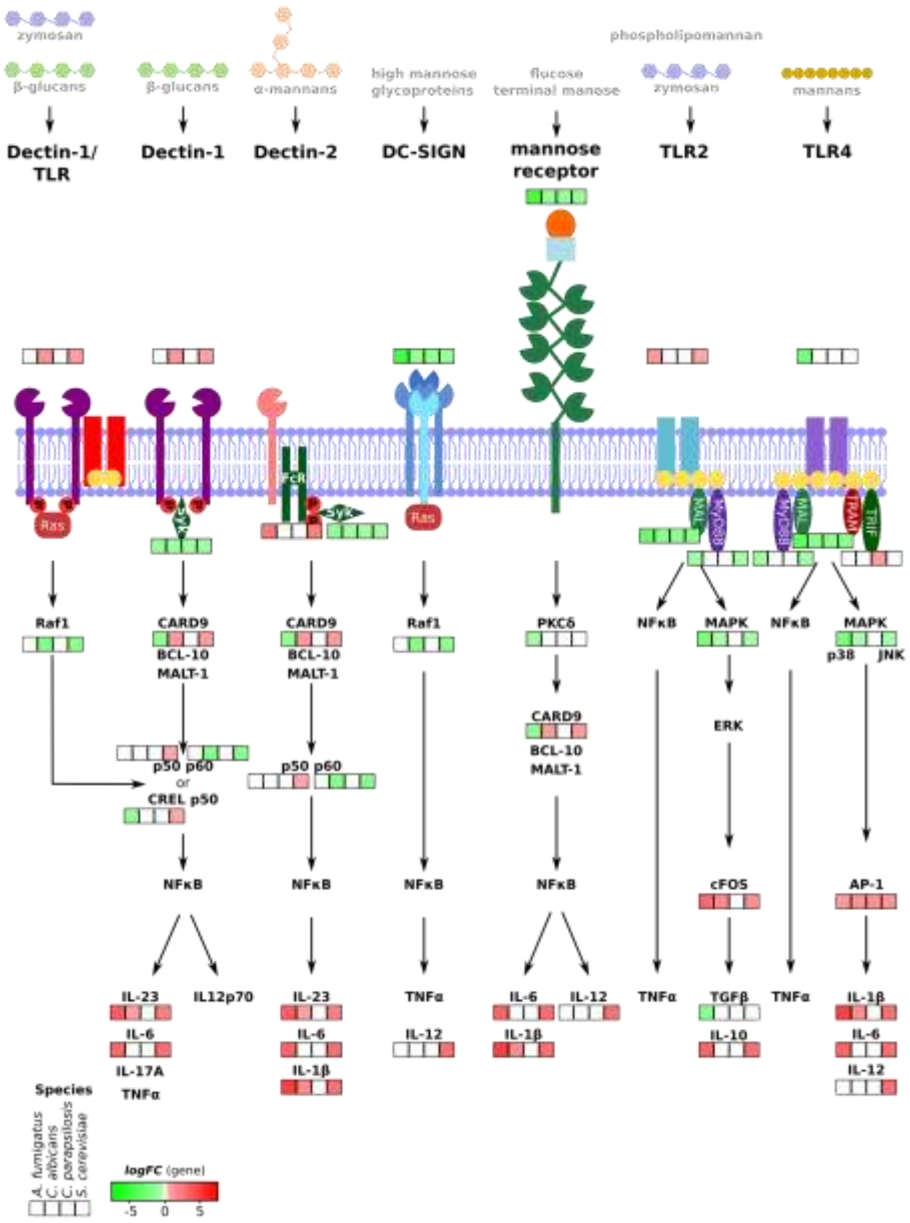

Figure S18

Known pathways involved in the DCs antifungal response to fungi triggered by endosomal receptors. The figure has been inspired by the schemes proposed by Ramirez-Ortiz and Means (Virulence 2012, 3:1-12) and Romani (Nature 2011, 11:275-288)

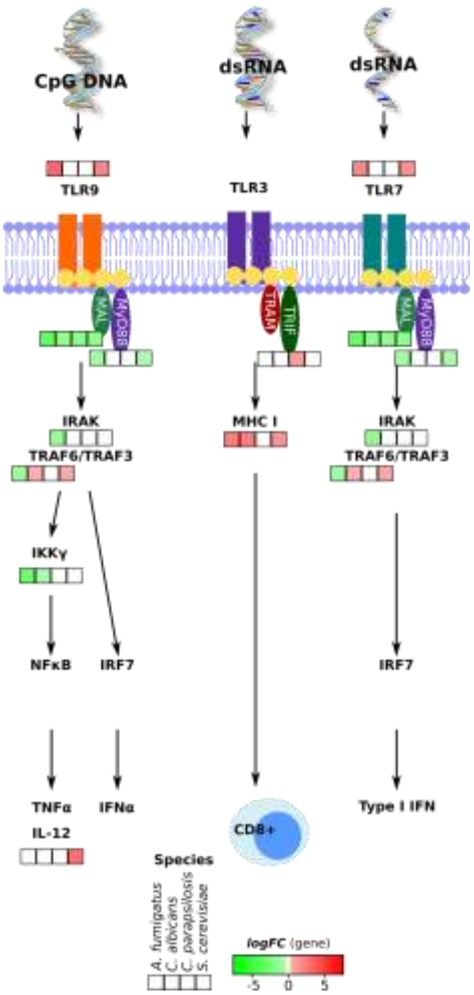

Supplement: Supplementary file 1 [file mic-13-282-s01.pdf]
